# Latent Inflammation and Insulin Resistance in Adipose Tissue

**DOI:** 10.1155/2017/5076732

**Published:** 2017-08-17

**Authors:** I. S. Stafeev, A. V. Vorotnikov, E. I. Ratner, M. Y. Menshikov, Ye. V. Parfyonova

**Affiliations:** ^1^Russian Cardiology Research and Production Centre, Moscow 121552, Russia; ^2^Faculty of Basic Medicine, M.V. Lomonosov Moscow State University, Moscow 119192, Russia; ^3^M.V. Lomonosov Moscow State University Medical Center, Moscow 119192, Russia; ^4^Endocrinology Research Centre, Moscow 117031, Russia

## Abstract

Obesity is a growing problem in modern society and medicine. It closely associates with metabolic disorders such as type 2 diabetes mellitus (T2DM) and hepatic and cardiovascular diseases such as nonalcoholic fatty liver disease, atherosclerosis, myocarditis, and hypertension. Obesity is often associated with latent inflammation; however, the link between inflammation, obesity, T2DM, and cardiovascular diseases is still poorly understood. Insulin resistance is the earliest feature of metabolic disorders. It mostly develops as a result of dysregulated insulin signaling in insulin-sensitive cells, as compared to inactivating mutations in insulin receptor or signaling proteins that occur relatively rare. Here, we argue that inflammatory signaling provides a link between latent inflammation, obesity, insulin resistance, and metabolic disorders. We further hypothesize that insulin-activated PI3-kinase pathway and inflammatory signaling mediated by several I*κ*B kinases may constitute negative feedback leading to insulin resistance at least in the fat tissue. Finally, we discuss perspectives for anti-inflammatory therapies in treating the metabolic diseases.

## 1. Introduction

According to the World Health Organization, obesity is observed in 30% of the world population [1]. It often precedes the type 2 diabetes mellitus (T2DM)—a complex state of metabolic disorders with a high rate of mortality worldwide. Insulin resistance and endothelial dysfunction are the specific features of T2DM. They lead to progression of cardiovascular diseases, which are at the top in the mortality rate. Thus, understanding molecular mechanisms of the obesity-associated insulin resistance is crucial for treating the metabolic and cardiovascular diseases.

Obesity is thought to arise when energy accumulation exceeds its consumption. Insulin is a key regulator of the carbohydrate and lipid metabolism. It plays a critical role in the conversion of energy imbalance into the obesity state. The main function of insulin is to increase glucose uptake from the blood. This is achieved in tissue-specific manner via increased translocation of the insulin-dependent glucose carrier GLUT4 in the skeletal muscle and fat cells [2, 3] and increased expression of GLUT1 in vascular endothelium cells [4]. The primary mechanism of obesity-induced insulin resistance has been demonstrated in the liver and skeletal muscle. It involves accumulation of diacylglycerol and activation of novel PKC isoforms PKC*ε* and PKC*θ* [5, 6]. However, this mechanism is hardly applicable to adipocytes that normally store lipid and contain large amounts of diacylglycerol. Therefore, it is likely that mechanisms of insulin resistance in fat cells differ from those in the liver and skeletal muscle.

At the cellular level, increased levels of free fatty acids and triglycerides were manifested by endoplasmic reticulum (ER) and oxidative stress, as well as hypertrophy of adipocytes and hypoxia due to impaired microcirculation and reduced oxygen supply. These conditions are ideal to trigger the inflammatory pathways leading to activation of I*κ*B kinase (IKK) and related kinases, which affect insulin signaling at its bottleneck by phosphorylating and switching off the function of insulin receptor substrate (IRS). Here, we review this mechanism and argue that anti-inflammatory agents have therapeutic potential towards insulin resistance.

## 2. Insulin Signaling in Health

The overall action of insulin is to increase energy storage in an organism in the form of transient glycogen and enduring fat. In addition to increasing glucose utilization form blood, insulin stimulates glucose conversion into stored glycogen in the skeletal muscle and liver, downregulates key gluconeogenic enzymes in the liver, and promotes accumulation of triglycerides by accelerating glucose degradation via glycolysis and synthesis of free fatty acids, as well as inhibiting lipolysis in the fat cells [7].

Insulin acts via cognate receptors on cells of the insulin-dependent tissues such as fat, liver, striated muscles, and endothelium [8–10]. It also binds, with a one order of magnitude less affinity, to insulin-like growth factor receptors in many other cells [11]. The signaling pathways activated by these receptors are similar, albeit not identical, which may account for differences in cell responses [12]. The interplay between these hormones is physiologically important for the progression of T2DM and metabolic dysfunction. However, it seems rather marginal to the development of insulin resistance [11] and for this reason will not be focused on here.

The insulin receptor is a heterotetrameric membrane glycoprotein consisting of two *α*- and two *β*-subunits. Insulin binds to the extracellular *α*-subunit and changes its conformation to promote ATP binding and autophosphorylation in the intracellular domain of the *β*-subunit, which is a tyrosine kinase. There are several autophosphorylation sites in the *β*-subunit. These sites form 3 groups according to their position either within active loop of the catalytic domain (Tyr-1158, Tyr-1160, and Tyr-1162), or juxtamembrane domain (Tyr-972), or distal C-terminal domain (Tyr-1328 and Tyr-1334). Phosphorylation of the active loop sites stimulates the kinase activity of insulin receptor, phosphorylation of juxtamembrane domain control receptor-substrate interactions, and stability of the receptor-substrate complex, whereas C-terminal phosphorylation mediates metabolic and mitogenic actions of insulin [13].

The insulin receptor substrate (IRS) proteins play a key role in insulin signaling. All of them are scaffolds that mediate further signaling by assembling and clustering specific signaling complexes via the Src homology (SH2) domain interactions [14]. The activated insulin receptor recruits IRS and phosphorylates it on tyrosine residues thus creating the binding sites for other signaling molecules containing the SH2 domains [15]. Thus, both the recruitment and binding of IRS to activated receptors and the subsequent binding of downstream effectors to IRS require tyrosine kinase activity of the receptor and phosphotyrosine-binding domains in the interacting proteins. Recruitment of IRS is further facilitated by its membrane localization mediated by the pleckstrin homology (PH) motif in IRS, which recognizes membrane-bound phosphoinositides. Thus, IRS proteins exert dual function by linking receptor-associated tyrosine kinase activity to cytoplasmic effectors and by bringing together the appropriate signaling molecules [16].

There are 6 isoforms of IRS. IRS-1 is the most common and widely expressed. The IRS-1 knockout mice have reduced growth and insulin action; however, the whole-body glucose tolerance remains normal [17]. The IRS-2 knockout mice exhibit stunted growth and development only in certain tissues, particularly in neurons and pancreatic islet cells [18]. They have impaired insulin signaling in the liver and dysfunction of islet cells; this combination results in diabetes. Expression of IRS-3, IRS-4, IRS-5, and IRS-6 is tissue-specific. In rodents, IRS-3 is present in adipocytes, the lung, and the liver [19], while in humans, the *IRS-3* is a pseudogene [20]. IRS-4 is found in the skeletal muscle, liver, heart, brain, and kidney [21]. The IRS-4 knockout mice display mild reduction in growth and glucose tolerance [22]. IRS-5 is expressed in the kidneys and liver; IRS-6 is found mainly in the skeletal muscle. IRS-5 and IRS-6 are the minor substrates of insulin receptor [23].

IRS proteins branch insulin signaling and allow formation of signaling networks. While PI3-kinase (PI3K) pathway mediates most of the metabolic effects of insulin, other pathways mediate mitogenic effects and terminate insulin signaling [8]. Independent of PI3K, IRS proteins link to adaptor proteins Grb2, Shc, Crk, Cbl, and tyrosine phosphatase SHP-2 [16]. Grb2 recruits the guanine nucleotide exchange factor SOS, which activates Ras GTPase and the Erk1/2 MAP-kinase pathway. It mediates cytoskeletal reorganization, proliferation, and differentiation responses to insulin [8]. Crk is a versatile adaptor; it redirects signals to p130Cas, a different scaffold protein that initiates cytoskeletal rearrangements [24]. Cbl is an E3 ubiquitin ligase common to many receptor tyrosine kinases; it controls their internalization and degradation in case of overactivation. The function of SHP-2 is to inactivate IRS by dephosphorylation, which allows IRS to escape degradation.

Metabolic effects of insulin downstream of IRS proteins are mediated by the class 1A PI3-kinases (PI3K). They are heterodimers of three catalytic subunits (p110*α*, p110*β*, and p110*δ*) and five p50–55/p85 regulatory subunits [25]. PI3Ks become recruited to tyrosine-phosphorylated IRS by virtue of their two SH2 domains in regulatory subunits. Subsequent activation involves deinhibition of the p110 catalytic subunit imposed by the regulatory one and binding of activated Ras GTPase to the Ras-binding domain (RBD) motif present in catalytic subunits. Given the membrane localization of the whole signaling module in the proximity of PI3K substrates and that Ras is concurrently activated by IRS, this results in full activation of PI3K. PI3K phosphorylates 3′-position in an inositol ring of phosphatidylinositol phospholipids, generating phosphatidylinositol-3,4-bisphosphate and phosphatidylinositol-3,4,5-trisphosphate (PIP3) [25].

**Figure 1:**
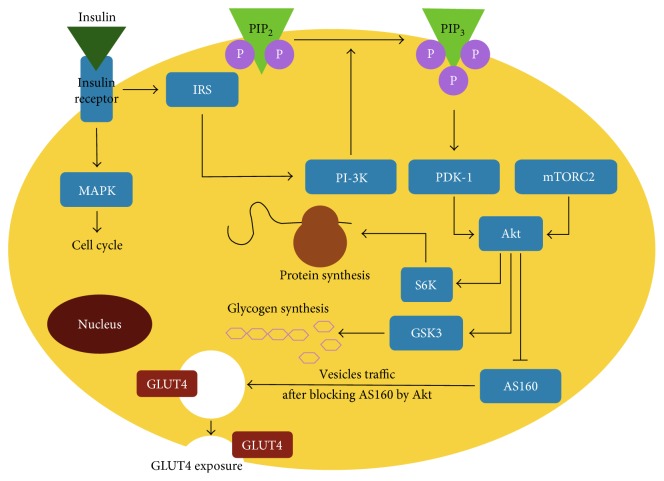
General scheme of insulin signaling in adipocytes with the main metabolic effects. AS160: Akt substrate 160 kDa; GSK3: glycogen synthase kinase type 3; IRS: insulin receptor substrate; MAPK: mitogen-activated protein kinase; mTORC2: mechanistic target of rapamycin; PI-3K: phosphatidylinositol-3-kinase; PDK-1: phosphoinositide-dependent kinase; S6K: ribosomal protein S6 kinase. According to Boucher et al. [15], with changes.

Signaling by PI3K involves protein-lipid interactions mediated by PH domains in the effector proteins. They recognize phosphoryl moieties on the sugar ring of phosphatidylinositol lipids. Despite having a common fold, PH domains are subtly different in the structure and variable in specificity. Some of them bind to phosphate at a certain position of inositol, regardless of whether and how many phosphates are in the other positions. Other PH domains prefer the phosphate in a different position of inositol. Typical PI3K effectors bear PH domains that recognize the 3′-phosphorylated inositol rings. These proteins mostly belong to the AGC family protein kinases (protein kinases A, G, and C as the founding members) [26]. Those involved in insulin signaling are the phosphoinositide-dependent kinase (PDK1), protein kinase B (PKB, a.k.a. Akt), p70 ribosomal protein S6 kinase (S6K1), serum and glucocorticoid-regulated kinase (SGK1), and atypical isoforms of protein kinase C (aPKC) that do not require Ca^2+^ and diacylglycerol for activation [15]. Both PDK1 and Akt contain a PH domain by which they bind to PIP3 on the membrane. Thereby, they are brought into proximity, and PDK1 phosphorylates Akt on Thr-308 (Figure 1) located in its activation loop [27]. Similarly, PDK1 serves as a master kinase to activate aPKC, S6K1, SGK1, and other AGC kinases [28]. To be effectively activated by PDK1, these kinases also require a docking site interaction. This site is created by additional phosphorylation within their hydrophobic motif. It recruits and activates PDK1, which then phosphorylates the activation loop of the effector kinases [29].

The hydrophobic motif site in Akt is Ser-473. In contrast to other AGC kinases, its phosphorylation is not obligatory for Akt activation; it is only needed for maximal catalytic activity [26]. It is achieved by a group of PI3K-related kinases including the mechanistic target of rapamycin (mTOR), DNA-dependent protein kinase (DNA-PK), and IKK. mTOR shuttles between two complexes, mTORC1 and mTORC2, that assemble in cells on different scaffold proteins Raptor and Rictor, respectively. Interrelationship between these two complexes is still not fully understood. Given a central role in the control of cell metabolism, proliferation, growth, survival, and aging, mTOR is likely subject to regulatory circuits and multifaceted signaling networks [30]. Thus, mTORC2 has been identified as a predominant Akt kinase [31], whereas mTORC1 is an indirect target of Akt. In addition to mTORC2, other kinases phosphorylate Ser-473 in Akt during stress responses. The DNA-PK is activated by double-strand breaks in DNA and phosphorylates Akt thus promoting survival in response to DNA damage [32].

Akt has many intracellular targets and mediates a large number of metabolic effects, including adipocytes (Figure 1). It regulates the glucose metabolism by phosphorylating the TBC1D4 protein, also known as AS160, and glycogen synthase kinase 3 (GSK3). AS160 is a GTPase-activating protein that inhibits activity of Rab10 and intracellular traffic of GLUT4-containing vesicles. Akt alleviates this inhibition, leading to increased translocation of Glut4 to the plasma membrane and increased glucose uptake, which is the central metabolic effect of insulin [15]. Akt also phosphorylates GSK3 in the liver and skeletal muscle and thereby activates synthesis and accumulation of glycogen [33]. In addition, insulin exerts Akt-independent effects on the glucose metabolism. It increases the activity of pyruvate dehydrogenase phosphatase thereby stimulating conversion of pyruvate to acetyl-CoA and also promotes activation of acetyl-CoA carboxylase by dephosphorylation thereby facilitating conversion of acetyl-CoA to malonyl-CoA and subsequent synthesis of fatty acids. Finally, insulin inhibits lipolysis and increases triglyceride content in adipocytes, both in the Akt-dependent and independent manner [7]. Altogether, these actions of insulin promote glucose utilization from the blood and energy storage through increased glycogenesis, synthesis of fatty acids, and fat deposition.

In addition, Akt mediates the adipogenic effects of insulin. Akt regulates mTORC1 [15], which activates the sterol regulatory element-binding protein (SREBP1). This is one of the major regulators of adipogenesis that activates transcription of *FABP4*, *ADIPOQ*, and *AGPAT2* genes [34]. Another pathway involves the forkhead box O (FOXO-1) transcription factor phosphorylated by Akt [35]. In the phosphorylated state, FOXO-1 is inactive and is translocated from the nucleus to cytoplasm, relieving the inhibition of peroxisome proliferator-activated receptor type *γ* (PPAR*γ*), the master regulator of adipogenesis. In parallel, phosphorylation of PPAR*γ* coactivator type 1*α* (PGC-1*α*) by Akt reduces the ability of PGC-1*α* to activate gluconeogenesis and oxidation of fatty acids, thus promoting development of the fat tissue [15, 36].

## 3. Feedback in Insulin Signaling as a Trigger of Disease

The uncontrolled activity of insulin cascade may cause metabolic dysregulation at both the cellular and whole body levels and lead to cancer development and other diseases [15, 37]. Therefore, insulin signaling is subject to fine regulation. This is achieved virtually at all steps of signal transduction and occurs by several means in cells. Thus, various lipids control recruitment and activation of the upstream components, such as insulin receptor, IRS, adaptor proteins, PI3K, and its target kinases; the phosphotyrosine and PH-domain interactions play a critical role. Feedback at these levels may involve uncoupling of the receptor and IRS, for example, by Grb10 and Grb14, inhibition of the receptor tyrosine kinase activity by supрressors of cytokine signaling (SOCS proteins), or obstructing activation of Akt the binding of Trb3 (Tribbles homolog 3) pseudokinases [15].

The most critical inhibitory mechanism of insulin signaling is Ser/Thr phosphorylation of IRS due to the negative feedback from the target molecules of insulin signaling. Multiple phosphorylated serines targeted by various Ser/Thr-kinases have been identified in different regions of IRS. These phosphorylations antagonize the effects of tyrosine phosphorylation of IRS that are needed for insulin signal transduction. The major kinases that target IRS are the stress-activated kinases (ERK, JNK, and AMPK), the inflammatory kinase IKK, and downstream kinase (Akt, atypical isoforms of PKC, mTOR, and S6K) [38]. The inhibitory effects of Ser/Thr phosphorylation are implemented by different ways (Figure 1), including dissociation of IRS from the insulin receptor, converting it to an inhibitor of the receptor tyrosine kinase activity, attenuation of tyrosine phosphorylation of IRS, increased degradation of IRS, or releasing it from the complexes with the adapter proteins [39, 40]. The common outcome is the lower cell response to insulin stimulation and insulin resistance when the feedback pathways maintain upregulated.

Dysregulation of insulin signaling often results in insulin resistance, the earliest feature in the pathogenesis of T2DM and metabolic disorders [41]. Genetic predisposition and metabolic dysfunction are the two general causes of T2DM. Recent estimates of T2DM heritability are varied from 25% to as much as 80% [42]. The molecular mechanisms of the acquired metabolic dysfunction are likely to involve the above-mentioned negative feedback and uncoupling of insulin signaling. What are the causes of this dysfunction and how and why they occur and develop into the disease state are the critical questions.

## 4. The Inflammatory Input

It is well understood that excessive calorie diet is the major cause of obesity in the modern society [43]. Facilitated by the low physical activity, excessive nutrients are converted into fat, which is the only long-term energy storage in an organism. Obesity promotes insulin resistance via ectopic lipid accumulation and PKC-related mechanism at least in the skeletal muscle [5]. This mechanism is doubtful in adipocytes because they always store large amounts of lipids that are not ectopic. However, excessive lipid accumulation stimulates hypertrophy and hyperplasia of adipocytes, as well as increased adipogenesis and recruitment of new cells [44]. In combination with a reduced blood supply, this creates hypoxic conditions and activates inflammatory signaling [45]. In addition, recruitment of adipose tissue macrophages (ATM) and their polarization into the M1 proinflammatory phenotype maintains inflammatory response, resulting in latent inflammation if overnutrition persists [46]. The inflammatory signals derived from the M1 macrophages promote insulin resistance [47], and inhibition of inflammatory signaling disturbs the link between obesity and insulin resistance [48, 49].

The latent inflammation appears to be critical to insulin resistance, turning the plain abdominal obesity with relatively low morbidity risk into T2DM and metabolic syndrome (as an association of obesity with T2DM and cardiovascular disease) [50]. The cellular mechanisms of latent inflammation coupled to activation of the nuclear transcription factor (NF-*κ*B) in adipose tissue have been extensively reviewed elsewhere [51–54]. Here, we only briefly mention them and focus on the link between inflammation and insulin signaling. As discussed below, there are several pathways of how inflammation may be coupled to insulin signaling in adipocytes, resulting in reduced cell responsiveness to insulin. Conversely, the insulin pathway may also target the inflammatory one, creating an inhibitory feedback that becomes active under conditions of overnutrition. Whereas the dominating concept has been that inflammatory kinase IKK*β* is critical to inflammatory link to insulin signaling, the studies over the recent years suggest that additional players are also involved and mediate reciprocal relationship between these phenomena.

**Figure 2:**
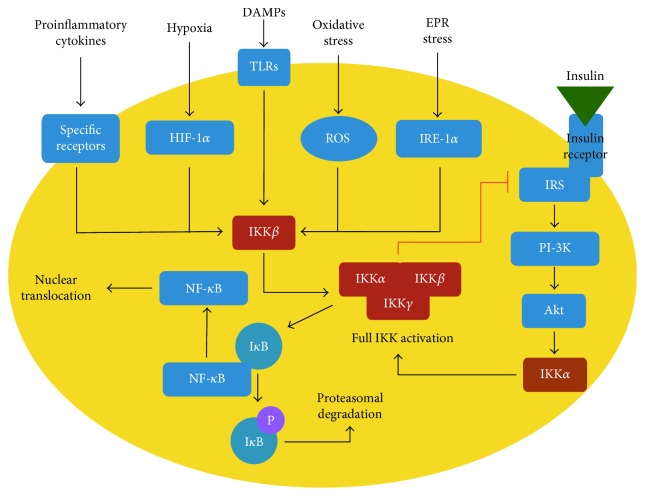
The inflammatory signaling with a focus on IKK and its interplay with insulin signaling. The red line symbolizes negative feedback. DAMPs: damage-associated molecular patterns; EPR stress: endoplasmic reticulum stress; HIF-1*α*: hypoxia inducible factor 1*α*; IRE-1*α*: inositol-requiring enzyme 1*α*; I*κ*B: inhibitory subunit of nuclear factor *κ*B; IKK: I*κ*B kinase; IRS: insulin receptor substrate; mTORC2: mechanistic target of rapamycin; NF-*κ*B: nuclear factor *κ*B; PI-3K: phosphatidylinositol-3-kinase; PDK-1: phosphoinositide-dependent kinase; ROS: reactive oxygen species; TLRs: toll-like receptors. According to Oeckinghaus et al. [55], with changes.

Inflammation plays an important role in the development of obesity, diabetes, metabolic syndrome, and many other diseases. The development of metabolic diseases can be viewed as a system where obesity-associated molecular pathologies (i.e., cellular hypoxia, ER stress, oxidative stress, and releasing of damage-associated molecular patterns (DAMPs)) are at the entrance, a number of metabolic dysfunctions (i.e., metabolic syndrome, diabetes, atherosclerosis, and arterial hypertension) are the outputs, and inflammatory process is a narrow bottleneck in between (Figure 2).

In adipocytes, free fatty acids appear to mediate the overnutrition effects on insulin signaling, as they do it in myocytes. However, in adipocytes, the coupling occurs via the inflammatory pathway rather than via PKC. NF-*κ*B is central to this mechanism. It controls expression of many genes critical to inflammation, apoptosis, cancer, viral infection, and autoimmune diseases.

Typically, latent inflammation prompts the proinflammatory macrophages to amass in the fat tissue. They secrete tumor necrosis factor-*α* (TNF*α*), which acts via two major mechanisms [56]. First, it binds the cognate TNF*α* receptors and activates NF-*κ*B signaling through death domain (DD) interactions with an adaptor molecule TRADD and TNF receptor-associated factors (TRAFs). Secondly, TNF*α* stimulates lipolysis in the fat cells [57]. The resultant free fatty acids are released from the apoptotic and necrotic adipocytes that increasingly appear in hypertrophied and stressed fat. They bind to Toll-like receptor (TLR) type 4 on adipocytes and activate NF-*κ*B inflammatory pathway in an autocrine manner [55]. In addition, interleukin-1 is released by the proinflammatory macrophages and activates NF-*κ*B pathway via the same TLR4 receptors [58].

In addition to IKK*β*, inflammatory response involves activation of JNK МАР-kinase that can act as a negative regulator of insulin signaling. Importance of JNK for development of the obesity-induced insulin resistance and latent inflammation in adipose tissue was first demonstrated by Hirosumi and colleagues in 2002, who found that JNK activity is significantly increased in the fat tissue of the high-fat diet and *ob*/*ob* mice in animal models of insulin resistance [59]. JNK may act through the alternative inflammatory transcription factor, AP-1. Activation of AP-1 accompanies expression of proinflammatory cytokines, such as TNF*α*, IL-1*β*, and others that stimulate cellular inflammatory responses in paracrine manner. Elimination of JNK1 results in decreased adiposity and significantly improved insulin sensitivity based on the blood glucose level, plasma insulin level, tyrosine phosphorylation of insulin receptor, and IRS.

Further research revealed two primary mechanisms mediated by JNK in adipose tissue. The first involves direct Ser/Thr phosphorylation of IRS-1 and inhibition of metabolic effects of insulin [60–62]. The second is mediated by JNK activation in ATM that can affect insulin signaling via different mechanisms. Classically, ATM polarize into M1-like phenotype and mediate paracrine activation of inflammatory and stress-activated kinases, such as JNK and IKK*β*, upregulation of inflammatory genes, and increased metabolic inflammation [63]. In addition, other immune cells (T-lymphocytes, dendritic cells, etc.) may also contribute to insulin resistance in adipocytes. Similar to ATM, they activate proinflammatory kinases, induce proinflammatory phenotypes of immune cells, and exert paracrine effects on adipocytes [64–66]. Intercellular communications are also likely to contribute, such as changes in insulin signaling in ATM can affect insulin sensitivity in adipocytes. Kawano et al. demonstrated that decreased signaling via PDK1/FOXO1 axis in myeloid cells leads to M1-like polarization of ATM and subsequent insulin resistance [67]. In summary, ATM and other immune cells induce inflammatory responses via both the canonical (JNK and IKK*β*) and auxiliary (paracrine) pathways. In this review, we focus on the classic inflammatory pathways and IKK biology in adipocytes.

Activation of inflammatory kinase IKK is a hallmark of NF-*κ*B signaling (Figure 2). This kinase phosphorylates the inhibitory protein I*κ*B, which binds to NF-*κ*B dimers and sequesters them in the cytoplasm. Phosphorylation promotes rapid ubiquitination and degradation of I*κ*B, thus liberating NF-*κ*B. It also exposes the nuclear localization signal on NF-*κ*B and allows NF-*κ*B to translocate to the nucleus and activate gene transcription [56, 68]. In the next section, we discuss IKK signaling in more detail and focus particularly on its interplay with insulin signaling in adipocytes.

## 5. IKK and Its Isoforms: Multiple Keys for Disease

A complexity of NF-*κ*B pathway stems from existence of several isoforms of IKK and I*κ*B that often act nonredundantly on an array of NF-*κ*B family members [58, 68]. They constitute two pathways that seem to be quite independent due to the use of different activating receptors and isoforms of signaling intermediates [69]. The canonical NF-*κ*B pathway regulates the innate immunity. It is activated by proinflammatory stimuli, such as TNF*α* and interleukin-1, and TLR agonists, such as bacterial antigen lipopolysaccharide (LPS) and free fatty acids. These receptors are coupled to activation of NEMO (IKK*γ*), which recruits and activates IKK*β*. The active complexes may or may not involve IKK*α*.

In contrast, the noncanonical pathway controls the adaptive immunity, modulates NF-*κ*B signaling, and limits NF-*κ*B activation at least in macrophages [70]. It is activated by a subset of TNF family members, such as B-cell activating factor (BAFF), CD40 ligand, and lymphotoxin *α*-*β* heterotrimers (LT*α*-*β*). They act via receptors linked to activation of NF-*κ*B-inducing kinase (NIK) (instead of NEMO in the canonical pathway) and IKK*α* (instead of IKK*β* therein) [69].

What gives a clue of how variety and flexibility of inflammatory signaling may be achieved is a substantial crosstalk between the two pathways, as well as involvement of multiple signaling isoforms [68]. The IKK proteins team up into active complexes (signalosomes) that phosphorylate I*κ*B. In the canonical pathway, the complexes consist of two catalytic subunits, IKK*α* and IKK*β*, which form homo- (*β*-*β*) or heterodimers (*α*-*β*), and a regulatory subunit, IKK*γ* (NEMO), that acts as scaffold. It positions the IKKs for efficient transautophosphorylation within the complex or brings them into proximity of the upstream kinases such as TAK1. In the noncanonical pathway, NIK activates the dimers of IKK*α* by phosphorylation [69].

Further downstream, the I*κ*B family includes classic I*κ*B proteins (I*κ*B*α*, I*κ*B*β*, and I*κ*B*ε*), NF-*κ*B precursors (p100 and p105), and nuclear I*κ*B proteins (I*κ*B*ζ*, Bcl-3, and I*κ*BNS) [58]. They display a strong preference to certain NF-*κ*B dimers, which are composed of RelA (p65), RelB, c-Rel, p50 (p105 precursor), p52 (p100 precursor), and Relish proteins by their homo- or heterodimerization. All these NF-*κ*B proteins have in common the Rel-domain that is responsible for dimerization. Thus, the variable composition of IKK complexes, their substrate I*κ*Bs, and NF-*κ*B targets may explain a great deal of signaling flexibility and a wide spectrum of outputs in response to multiple external stimuli picked up by NF-*κ*B pathway. In addition to differences in composition, the canonical and noncanonical pathways are functionally interconnected. They influence each other's signal dynamics, which is important to determine a type of the cell response [68].

Insulin cascade is one of the IKK targets. IKK*β* has been well documented to link inflammatory and insulin pathways in different cells [71] including adipocytes [72]. Once activated, IKK*β* sets up a crosstalk by phosphorylating IRS proteins at serine residues. This phosphorylation has been demonstrated in adipocytes in vitro [73], indicating that IKK*β* contributes to insulin resistance by attenuating the insulin signaling immediately at the postreceptor level. In the same vein, IKK*γ* (NEMO) has been reported to mediate phosphorylation of IRS on typical inflammation-responsive Ser307 [74]. These results added to the *in vivo* findings that obesity- and diet-induced insulin resistance is restored in mice with targeted disruption of IKK*β* [72]. Consistent with the proposed role of neutrophils and macrophages in inflammation, conditional knockout of IKK*β* in myeloid precursors of these cells led to increased overall sensitivity to insulin [72]. Thus, the link between inflammatory and insulin pathways has been established and suggested a role of inflammation in the development of insulin resistance in adipocytes.

Evidence has been accumulated that, in addition to IKK*β*, other IKKs are also involved in inflammatory signaling related to inflammation-induced insulin resistance. The early study has implicated IKK*α* in TNF*α*-induced NF-*κ*B activation [75]. Concurrently, Ozes et al. found that Akt phosphorylates IKK*α* on unique Ser23 and contributes to NIK-dependent activation of NF-*κ*B by TNF*α* in a variety of cell lines [76]. It was almost immediately questioned by Delhase et al., who reported a lack of the effect of Akt on NF-*κ*B activation in HeLa cells treated by TNF*α* or insulin-like growth factor-1 [77]. This prompted a new research to clarify how PI3-kinase/Akt axis activates NF-*κ*B. It showed that this activation is cell-specific and is determined by the expression ratio of IKK*α*/IKK*β*, so that PI3K inhibition can fully inhibit, partially impair, or minimally affect the NF-*κ*B response to TNF*α* [78]. This means that PI3K/Akt would have negligible effects on NF-*κ*B activation in HeLa cells that express traces of IKK*α* but may profoundly activate NF-*κ*B in adipocytes, which express considerable amounts of IKK*α* [78]. To date, many reports confirmed the involvement of PI3K/Akt in activation of both the canonical and noncanonical NF-*κ*B pathways [79–81], role of IKK*α* [70, 82], and identified additional players such as Raptor/mTOR [83] and Rictor/mTOR [84]. However, bearing in mind that IKK*α* is shared by the canonical and noncanical NF-*κ*B pathways and needed for activation of IKK*β* [69], these studies are consistent with the general concept that canonical IKK*β* mediates the inflammatory input to insulin resistance. Rather, these studies underpin the complexity of the link and identify new potential players, such as PI3K/Akt and IKK isoforms.

To add the complexity, novel IKK-related kinases, IKK*ε* (also known as IKK-i) and TBK1, have been implicated in obesity- and inflammation-induced insulin resistance [85, 86]. These kinases specifically mediate induction of interferon-1 expression and antiviral response [87]. In contrast to other IKKs, IKK*ε* is not ubiquitous being preferentially expressed in the pancreas, thymus, spleen, peripheral blood leukocytes, and placenta [88]. However, the IKK*ε* levels are substantially increased in the liver and white adipose cells of the high-fat diet- (HFD-) fed mice, as compared to those of IKK*α* and IKK*β* [85]. The IKK*ε* knockout mice are protected from chronic, diet-induced inflammation, have preserved insulin signaling, and improved energy homeostasis [85]. Remarkably, a follow-up study reported the finding of amlexanox as a high-affinity inhibitor of IKK*ε* and TBK1, but not of the IKK*α* or IKK*β* [86]. Amlexanox produced similar effects on HFD-fed mice to those observed in IKK*ε* knockout mice, suggesting that IKK*ε* mediates obesity- and inflammation-induced insulin resistance in adipocytes, whereas IKK*α*/IKK*β* may have a lesser role.

The idea that feedback mechanisms contribute to insulin resistance is highly applicable to inflammatory input. Insulin ultimately triggers PI3K/Akt pathway, which contributes to IKK/NF-*κ*B activation. Thereby, insulin may initiate negative feedback to IKK-mediated phosphorylation of IRS and inhibition of insulin signaling. It is long known that insulin activates NF-*κ*B in mammalian cells [89]. Hyperinsulinemia also increases NF-*κ*B activity in vascular smooth muscle cells that are physiologically liable to insulin action [90]. Whether the feedback occurs in the insulin-targeted tissues (i.e., fat, liver, and skeletal muscle) and becomes stable to result in insulin resistance depends on sustained local activation of IKK. Given the potential role of macrophages in maintaining latent inflammation, they are likely to play a role in increased activity of IKK/NF-*κ*B pathway. Future research should bring critical information about physiological site of their action. It may be located in the fat tissue and propagated to other tissues by free fatty acids or other means. Alternatively, the resident macrophages may deliver the inflammatory input locally. In any case, IKK isoforms and interplay between the canonical and noncanonical NF-*κ*B pathways will be important.

Finally, it has to be noted that PI3-kinase signaling is also mediated by functionally different Akt isoforms. While Akt1 mediates insulin effects on protein synthesis and proliferation, the Akt2 isoform apparently mediates insulin effects on glucose metabolism [91]. The possibility of whether the Akt1 isoform activates IKK*α* to create inflammatory feedback to insulin resistance mediated by Akt2 is plausible, but unknown. Alternatively, phosphorylation of IKK*α* by Akt may not be the only means to activate stable feedback to insulin resistance. The macrophage-derived TNF*α* activates the atypical and novel PKC isoforms (PKC*ζ* and PKC*θ*, resp.). They both can phosphorylate IKK*β* and activate inflammatory response leading to insulin resistance in the fat tissue [92].

## 6. Anti-Inflammatory Therapeutic Applications

The concept of anti-inflammatory approach in modern diabetology was conceived by the fundamental work of Hotamisligil et al. [93], who demonstrated the role of TNF in the development of insulin resistance in the animal model of a high-fat diet. The first clinical trial with the use of TNF antagonists was initiated in 1996 when a TNF-specific antibody was used as the first drug aimed at blocking the inflammatory action of TNF. Perhaps, a few number of patients (10 patients) and a single administration of antibody did not allow to reveal significant effects on insulin sensitivity [94]. The significant effects were only achieved if the soluble TNF receptor-Fc fusion protein (etanercept) was used in a regular administration for 4 weeks [95, 96]. Subsequent study showed that administration of etanercept during 26 weeks significantly decreases the fasting blood glucose level and increases the blood level of high molecular weight adiponectin [97]. However, the results of this study should be taken with caution, since the sample was made from prediabetic patients [98].

Substantiating the fundamental link between inflammation and insulin resistance, further research has discovered a role of IKK beta in the formation of insulin resistance [99]. In the parallel clinical studies, salsalate (salicylate) has been used as the classical anti-inflammatory drug. The longest studied tested the use of salsalate for the patients with T2DM during 12 weeks [100, 101] or 48 weeks [102]. All these studies demonstrated reduced blood levels of fasting blood glucose, glycated hemoglobin [100, 102], and triglycerides [100, 102], while the level of high molecular weight adiponectin was increased [101, 102].

The majority of current anti-inflammatory drugs that exert clinical sound effects are the antagonists of proinflammatory cytokines. The proinflammatory cytokine IL-1 beta and its receptor were targeted to combat insulin resistance. The longest clinical studies using an IL-1 receptor blocker (anakinra) spanned 13 [103] and 39 weeks [104]; they showed significantly decreased level of C-reactive protein and increased insulin secretion. A monoclonal antibody against IL-1 beta (canakinumab) has been also continuously administered for 16 weeks [105] and demonstrated decreased blood levels of C-reactive protein and glycated hemoglobin. It also tended to increase insulin secretion.

Still, many anti-inflammatory approaches await clinical application. Among possible targets are MCP-1 (monocyte chemoattractant protein type 1) [106], regulators of the epigenetic state of cells (sirtuins) [107], regulators of the synthesis of proinflammatory mediators (12-lipoxygenase, 15-lipoxygenase, etc.) [108], and others. However, no anti-inflammatory drugs specific to inflammatory kinase IKK or its isoforms are currently used. The only exception is the nonspecific inhibitor of the NF-*κ*B signaling salsalate, which has not demonstrated significant clinical effects on its own. By contrast, drugs acting on the key downstream components of the inflammatory pathways, such as IKK, are currently missing. This calls into a need for fundamental research of the role of different IKK isoforms in coupling the insulin and inflammatory pathways in search of novel-specific anti-inflammatory drugs with antidiabetic action.

## 7. Conclusions and Future Directions

Here, we examined basic mechanisms of insulin resistance in the fat tissue with a focus on inflammatory pathways. Evidence is accumulated that inflammatory signaling is complex. It is nonredundantly mediated by multiple isoforms of signaling proteins, including the IKK kinases that link inflammation to insulin signaling. All of the latter (IKK*α*, IKK*β*, IKK*γ*, and IKK-related kinases IKK*ε* and TBK1) appear to be involved. As a result, insulin signaling becomes uncoupled by IKK-mediated phosphorylation of IRS. On the other hand, PI3-kinase and Akt mediate the crosstalk from insulin signaling to inflammatory pathways, resulting in their activation. The crosstalk activity may thereby generate a negative feedback loop leading to insulin resistance and T2DM if maintained. In obesity, proinflammatory macrophages are capable of maintaining the crosstalk activity either from adipose depots or in local environments. The Akt isoform diversity likely contributes to the feedback. Other players, such as atypical/novel PKC isoforms, are also expected to be involved.

An obvious question is whether we can combat insulin resistance and T2DM by interfering with the inflammatory input. Such an approach is currently paid much experimental attention, and favorable results are obtained in many conditions tested (recently reviewed by Esser et al. [109], Lackey and Olefsky [54], and Shoelson et al. [110]). However, the clinical studies with respect to human T2DM are rather limited. The tested strategies include the use of general anti-inflammatory drugs, antibodies to inflammatory cytokines, thiazolidinediones, inhibitors of macrophage recruitment into metabolic tissues, and dietary approaches.

Aspirin has been for years used in patients with T2DM as a cyclooxygenase inhibitor and an anti-inflammatory agent. However, if used in the doses needed to substantially affect insulin resistance, it has serious side effects including gastrointestinal bleeding. Salicylate, aspirin metabolite, and especially its dimer, salsalate, have much less side effects. Though promising at the beginning, they brought rather conflicting results ranging from modest to no effects on glycaemia and insulin sensitivity. The most encouraging data are brought by antibody-based therapies against individual cytokines, such as TNF*α* and interleukin-1 and interleukin-6, and especially interleukin-1*β*. Notably, this approach aims at removing the primary cause of increased inflammation; however, an issue of targeting a particular tissue perhaps will be on the agenda. Thiazolidinediones (glitazones), such as rosiglitazone and pioglitazone, have been widely used as insulin sensitizers and activators of PPAR*γ*. However, taking into account a key role of PPAR*γ* in adipogenesis and cardiovascular side effects of these drugs, their use has been restricted. The therapies that prevent recruitment of macrophages and neutrophils into sites of metabolic inflammation have a potential but are at the beginning. Yet, their use may be complicated by a wide spectrum of substances with chemotactic activity and a long list of their receptors on these cells. An additional concern comes from the ability of macrophages to develop either pro- or anti-inflammatory phenotype, which may locally counteract inflammation. Finally, the dietary fish-oil supplementations and purified *ω*-3 unsaturated fatty acids demonstrated modest results and now are far from developing into therapeutics.

As for perspectives, the major concern of interfering with the key intracellular components of immune signaling, such as TNF*α* receptors or ΙΚΚ*β*, would be expectancy of critical side effects on immune responses. On the other hand, remarkable complexity of the immune signaling and NF-*κ*B pathways calls for development of broad therapeutic strategies through combination of anti-inflammatory approaches. Rather, mild interventions that simultaneously target few secondary components may be as effective as individual targeting of the key components but may provide less side effects. Surely, this will require much deeper knowledge of organization and coordinate function of intracellular immune system and its signaling components.

## Abbreviations

ATM: Adipose tissue macrophages
AMPK: AMP-dependent kinase
AP-1: Activator protein type 1
AS160: Akt substrate 160 kDa
aPKC: Atypical isoforms of protein kinase C
BAFF: B-cell activating factor
DNA-PK: DNA-dependent protein kinase
DAMPs: Damage-associated molecular patterns
DD: Death domain
ER: Endoplasmic reticulum
ERK: Extracellular signal-regulated kinase
FOXO-1: Forkhead box O
GLUT4: Glucose transporter type 4
Grb2: Growth factor receptor-bound protein type 2
GSK-3: Glycogen synthase kinase type 3
HFD: High-fat diet
HIF-1*α*: Hypoxia inducible factor type 1*α*
IKK: I*κ*B kinase
IRS: Insulin receptor substrate
IRE-1*α*: Inositol-requiring enzyme 1*α*
iNOS: Inducible NO synthase
I*κ*B: Inhibitory subunit of NF-*κ*B
IL-1*β*: Interleukin type 1*β*
I*κ*BNS: NF-*κ*B inhibitor delta
JNK: c-Jun N-terminal kinase
LPS: Lipopolysaccharide
LT*α*-*β*: Lymphotoxin *α*-*β*
MAP-kinase: Mitogen-activated protein kinase
mTOR: Mechanistic target of rapamycin
NF-*κ*B: Nuclear factor *κ*B
NIK: NF-*κ*B-inducing kinase
PKC: Protein kinase C
PI3K: Phosphatidylinositol 3′-kinase
PIP3: Phosphatidylinositol-3-phosphate
PDK-1: Phosphoinositide-dependent kinase type 1
PKB (Akt): Protein kinase B
PH-domain: Pleckstrin homology domain
PPAR*γ*: Peroxisome proliferator-activated receptor type *γ*
PGC-1*α*: PPAR*γ* coactivator type 1*α*
PKA: cAMP-dependent protein kinase
RBD: Ras-binding domain
ROS: Reactive oxygen species
SH2: Src homology type 2 (domain)
SOS: Son of sevenless
SHP-2: SH2 domain-containing phosphatase
S6K: p70 S6 kinase
SGK: Serum and glucocorticoid-regulated kinase
SREBP1: Sterol regulatory element-binding protein
SOCS: Suppressors of cytokine signaling
T2DM: Type 2 diabetes mellitus
Trb3: Tribbles homolog 3
TNF*α*: Tumor necrosis factor-*α*
TRAF: TNF*α* receptor-associated factor
TLR: Toll-like receptor
TBC1D4: TBC1-domain family member 4.
